# Hepatic steatosis in humans is associated with preserved glucagon action on amino acid metabolism

**DOI:** 10.1172/JCI200913

**Published:** 2025-12-23

**Authors:** Hannah E. Christie, Sneha Mohan, Aoife M. Egan, Federica Boscolo, Chiara Dalla Man, Scott M. Thompson, Michael Jundt, Chad J. Fleming, James C. Andrews, Kent R. Bailey, Michael D. Jensen, K. Sree Nair, Adrian Vella

**Affiliations:** 1 Division of Endocrinology, Diabetes, Metabolism and Nutrition, Mayo Clinic, Rochester, Minnesota, USA.; 2 Department of Information Engineering, University of Padova, Padova, Italy.; 3 Division of Vascular and Interventional Radiology and; 4 Division of Biomedical Statistics and Informatics, Mayo Clinic, Rochester, Minnesota, USA.

**Keywords:** Endocrinology, Metabolism, Amino acid metabolism, Glucose metabolism, Insulin

## Abstract

**BACKGROUND:**

Amino acid (AA) concentrations are increased in prediabetes and diabetes. Since AAs stimulate glucagon secretion, which should then increase hepatic AA catabolism, it has been hypothesized that hepatic resistance (associated with hepatic fat content) to glucagon’s actions on AA metabolism leads to hyperglucagonemia and hyperglycemia.

**METHODS:**

To test this hypothesis, we therefore studied lean and obese individuals, the latter group with and without hepatic steatosis as defined by proton density fat fraction (PDFF) > 5%. After an overnight fast, femoral vein, femoral artery, and hepatic vein catheters were placed. [3-^3^H] glucose and l-[1-^13^C,^15^N]-leucine were used to measure glucose turnover and leucine oxidation, respectively. During a hyperglycemic clamp, an AA mixture was infused together with insulin and glucagon (1.5 ng/kg/min 0–120 minutes; 3.0 ng/kg/min 120–240 minutes). Tracer-based measurement of hepatic leucine oxidation in response to rising glucagon concentrations and splanchnic balance (measured using arteriovenous differences across the liver) of the other AAs were the main outcomes measured.

**RESULTS:**

The presence of hepatic steatosis did not alter hepatic glucose metabolism and leucine oxidation in response to insulin and rising concentrations of glucagon. Splanchnic balance of a few AAs and related metabolites differed among the groups. However, across-group differences of AA splanchnic balance in response to glucagon were unaffected by the presence of hepatic steatosis.

**CONCLUSION:**

The action of glucagon on hepatic AA metabolism is unaffected by hepatic steatosis in humans.

**TRIAL REGISTRATION:**

Clinical Trials.gov: NCT05500586.

**FUNDING:**

NIH National Institute of Diabetes and Digestive and Kidney Diseases DK116231, DK78646, DK116231, DK126206, and DK116231.

## Abstract 2

Hepatic resistance to glucagon’s actions on amino acid catabolism is not a significant factor in postprandial metabolism regardless of obesity or hepatic steatosis.

## Graphical abstract

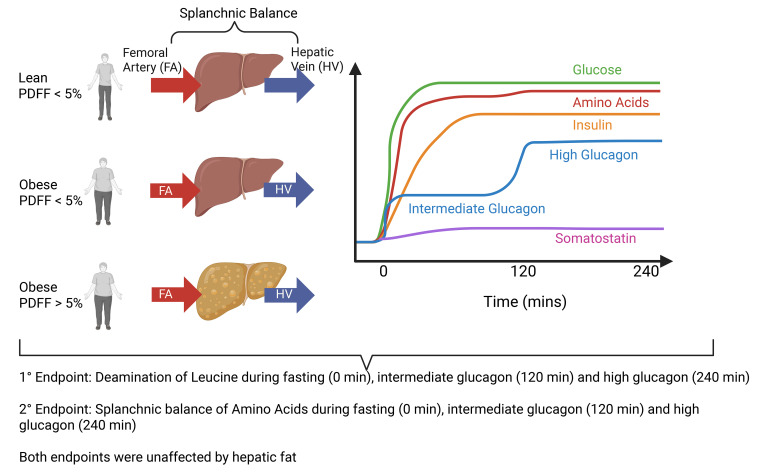

## Introduction

It is estimated that 35%–40% of the adult US population has obesity (1). Obesity-related metabolic dysfunction–associated steatotic liver disease (MASLD) is reported to have a prevalence of 24% (2). Both obesity and MASLD (3–5) independently increase the risk of type 2 diabetes (T2DM), a disease that increases morbidity and mortality while costing more than $170 billion per year (6), making prevention important. T2DM is characterized by insulin secretion that is inadequate for the prevailing insulin action and by glucagon concentrations inappropriate for the prevailing hyperglycemia. α cell dysfunction (7), elevated fasting amino acids (AAs) (8), and AA metabolites, e.g., α-aminoadipic acid (9), are markers of T2DM risk.

In rodents, impaired hepatic glucagon signaling results in hyperglucagonemia and increased α cell mass — an effect mediated by increases in circulating AA concentrations (10). Thus, the elevated concentrations of branched-chain amino acid (BCAA) and other AA metabolites that arise from impaired glucagon signaling (8) would further contribute to α cell dysfunction. If impaired glucagon-induced hepatic AA catabolism is the cause of hyperglycemia in MASLD, this would require selective preservation of the effects of glucagon on hepatic glucose metabolism (11). This could be explained by the different signaling pathways for glucagon actions on AA as opposed to other macronutrients (12, 13).

Recent reports suggest that obese humans with MASLD are resistant to the acute stimulation of hepatic AA catabolism by glucagon (14, 15). This is important because α cells respond to elevated AA concentrations (16) by secreting glucagon (17, 18). A failure to stimulate hepatic AA clearance could result in glucagon secretion inappropriate for the prevailing glucose concentrations. In a prior human study addressing this question, most participants with obesity also had MASLD such that it is unclear if the failure of glucagon to stimulate AA clearance and ureagenesis was caused by obesity, hepatic steatosis, or both (14). There are other technical limitations to this prior experiment that may affect the generalizability of its conclusions (see Discussion).

Previously, we have shown that α cell dysfunction occurs early in the pathogenesis of prediabetes and predicts a longitudinal decline in glucose tolerance (7, 19). It is possible that rather than being due to intrinsic α cell dysfunction, this is an appropriate response to (abnormally elevated) AA. AA concentrations in the circulation represent a balance between appearance (whether from endogenous sources or ingestion) and clearance. To accurately measure hepatic extraction of AAs under fasting and postprandial conditions, we studied patients after placement of the appropriate intravascular catheters to facilitate measurement of AA arteriovenous differences across the liver.

To specifically address whether the presence of hepatic fat alters the effects of glucagon on hepatic AA metabolism, we studied lean and obese individuals in whom hepatic fat was quantified using MRI. In this way we were able to recruit obese individuals with a wide range of hepatic fat content. Patients were studied after an overnight fast, when femoral vein, femoral artery, and hepatic vein catheters were placed (Supplemental Figure 1; supplemental material available online with this article; https://doi.org/10.1172/JCI200913DS1). Insulin was infused at 0.8 mU/kg/min to mimic postprandial conditions, while peripheral glucose concentrations were maintained at approximately 9.5 mmol/L. Glucagon was infused at 2 concentrations (“intermediate” and “high”) to mimic early postprandial concentrations observed after ingestion of a high-protein meal. A mixture of AAs, used clinically for total parenteral nutrition, was also infused to mimic postprandial conditions.

We report that while hepatic steatosis was associated with impaired insulin action, there was no effect on hepatic glucose metabolism in response to rising glucagon concentrations. Tracer-based measurement of leucine oxidation in response to rising glucagon concentrations showed no effect of obesity alone or obesity with hepatic steatosis on leucine metabolism in the liver. Subtle differences in the splanchnic balance of some AA and related metabolites were identified and, in a few examples, correlated with hepatic fat content. However, the response to glucagon did not differ significantly across groups. Overall, these data suggest that hepatic glucagon resistance does not play a major role in the handling of AA by the liver.

## Results

### Patient characteristics.

A total of 20 patients were studied. By design, patients in the obese group had higher total body weight and BMI than the lean patients (Table 1). The subgroup recruited because of the presence of high hepatic fat had a PDFF > 5%, which, again by design, differed significantly from that in the other 2 groups. The increase in hepatic fat content was not accompanied by any differences in hepatic stiffness or liver iron content. Fasting glucose concentrations and HbA1c values were higher in the groups with obesity but did not differ in those with and without increased hepatic fat. Despite increased Φ, compared with the lean group, when expressed as a function of the prevailing *S_i_*, DI was decreased in patients with obesity. These indices, estimated by the oral minimal model (20), did not differ in the patients with obesity with and without increased hepatic fat.

### Concentrations of glucose, insulin, C-peptide, and glucagon during fasting and then during the hyperglycemic clamp.

**Figure 1:**
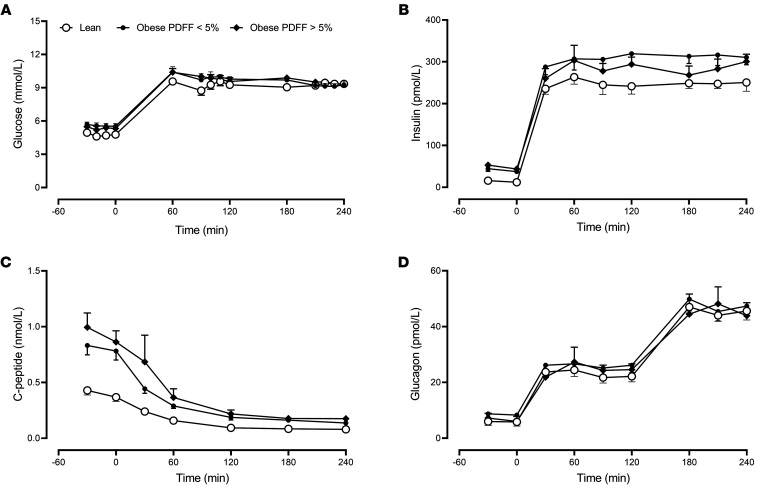
Glucose, insulin, C-peptide, and glucagon concentrations during the experiment. The mean (± SEM) glucose (**A**), insulin (**B**), C-peptide (**C**), and glucagon (**D**) concentrations during fasting and then during the hyperglycemic clamp, for lean patients (○), obese patients with a PDFF < 5% (●), and obese patients with a PDFF > 5% (♦). PDFF, proton density fat fraction. *n* = 7 in the lean group, *n* = 6 in the obese group with PDFF < 5%, and *n* = 7 in the obese group with PDFF > 5%.

Prior to the start of the clamp, fasting glucose concentrations did not differ significantly among the groups (Figure 1A: 4.8 ± 0.3 vs. 5.6 ± 0.2 vs. 5.4 ± 0.02 mmol/L, lean vs. obese PDFF < 5% vs. obese PDFF > 5%, respectively, *P* = 0.07). By design, glucose concentrations during the clamp did not differ among the groups during intermediate and high glucagon infusion (120 and 240 minutes, respectively — Figure 1A).

Fasting insulin concentrations were significantly higher in patients with obesity (Figure 1B: 14 ± 1 vs. 41 ± 5 vs. 48 ± 7 pmol/L, *P* < 0.01). There was no difference in fasting insulin concentrations in obese patients with and without increased hepatic fat. Insulin concentrations during the clamp did not differ between groups (*P* = 0.07 — Figure 1B).

Fasting C-peptide concentrations mirrored the differences in fasting insulin concentrations (Figure 1C: 0.40 ± 0.04 vs. 0.81 ± 0.08 vs. 0.93 ± 0.11 nmol/L, *P* < 0.01) and remained slightly, but significantly (*P* < 0.01), higher in the patients with obesity after the initiation of somatostatin infusion during the clamp.

Glucagon concentrations prior to the initiation of the clamp did not differ between groups (Figure 1D). At 120 minutes glucagon infused at 1.5 ng/kg/min (intermediate) resulted in concentrations (22 ± 2 vs. 26 ± 2 vs. 24 ± 2 pmol/L) that did not differ between groups (*P* = 0.42). At 240 minutes glucagon infused at 3.0 ng/kg/min (high) resulted in concentrations (45 ± 2 vs. 48 ± 3 vs. 46 ± 5 pmol/L) that also did not differ between groups (*P* = 0.86 — Figure 1D).

### Rates of glucose infusion, glucose disappearance, and endogenous glucose production during fasting and then during the hyperglycemic clamp.

**Figure 2:**
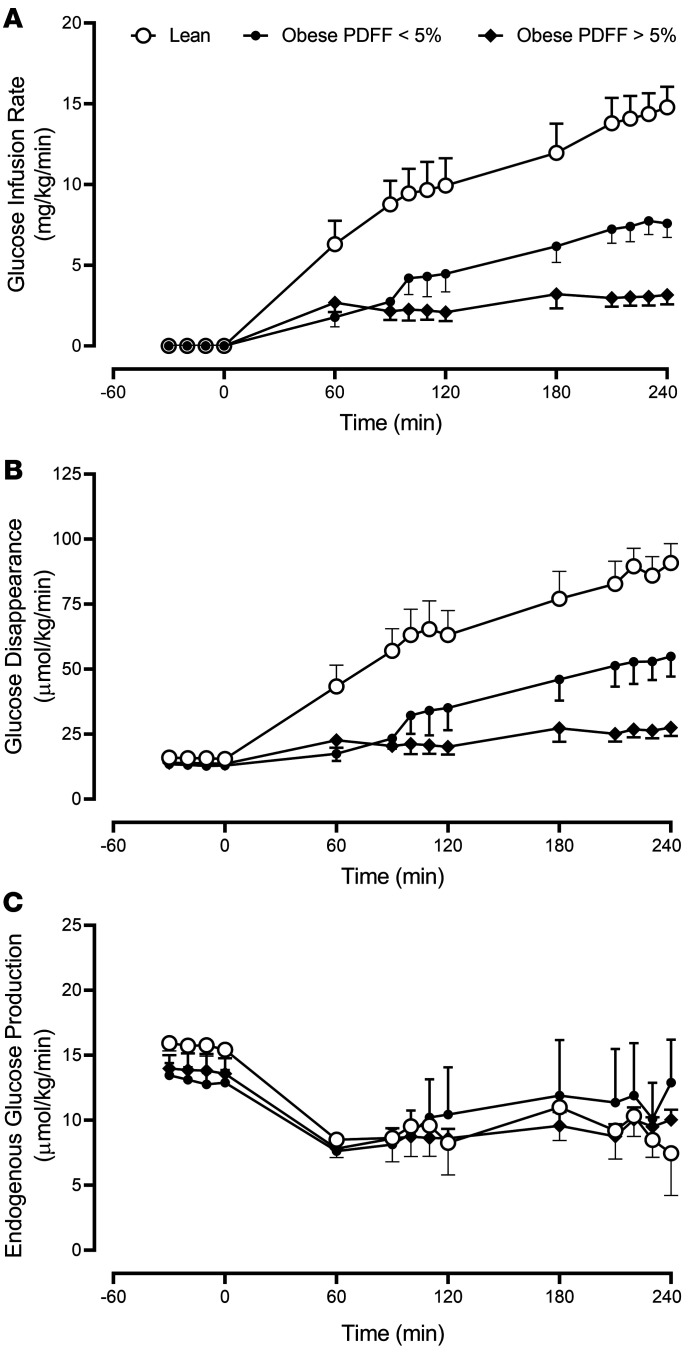
Glucose infusion rate, glucose disappearance, and endogenous glucose production during the experiment. The mean (± SEM) glucose infusion (**A**), glucose disappearance (**B**), and endogenous glucose production (**C**) rates during fasting and then during the hyperglycemic clamp, for lean patients (○), obese patients with a PDFF < 5% (●), and obese patients with a PDFF > 5% (♦). PDFF, proton density fat fraction. *n* = 7 in the lean group, *n* = 6 in the obese group with PDFF < 5%, and *n* = 7 in the obese group with PDFF > 5%.

The amount of glucose infused to maintain the hyperglycemic clamp was greater in the lean patients compared with those with obesity (*P* < 0.01 — Figure 2A). During intermediate glucagon infusion (90 to 120 min), there were no significant differences in the glucose infusion rate (GIR) between obese patients with and without increased hepatic fat. However, during the high glucagon infusion (210 to 240 min), post hoc testing showed significant differences in GIR between the 2 groups with obesity (Figure 2A: 7.5 ***±*** 0.8 vs. 3.1 ± 0.5 mg/kg/min, obese PDFF < 5% vs. obese PDFF > 5%, respectively, *P* = 0.02).

The rate of glucose disappearance (Rd) exhibited a similar pattern to GIR with no differences observed in the fasting state (–30 to 0 min) among the 3 groups (Figure 2B: *P* = 0.15). However, Rd was higher in the lean patients compared with those with obesity (*P* < 0.01 — Figure 2B) during the clamp. During the high glucagon infusion (210 to 240 min), post hoc testing showed significant differences in Rd between the 2 groups with obesity (Figure 2B: 53 ± 7 vs. 26 ± 3 μmol/kg/min, obese PDFF < 5% vs. obese PDFF > 5%, respectively, *P* = 0.02).

Rates of endogenous glucose production (EGP) during the fasting state did not differ between groups (Figure 2C: 15.7 ± 0.6 vs. 13.1 ± 0.9 vs. 13.8 ± 1.2 μmol/kg/min, lean vs. obese PDFF < 5% vs. obese PDFF > 5% respectively, *P* = 0.15). Also, no differences in EGP were apparent during the intermediate (*P* = 0.88) and high (*P* = 0.57) glucagon infusion rates.

### Net splanchnic glucose balance, splanchnic extraction ratio, splanchnic glucose uptake, and splanchnic glucose production during fasting and then during the 2 stages of the hyperglycemic clamp.

Net splanchnic glucose balance (NSGB), splanchnic extraction ratio (SER), splanchnic glucose uptake (SGU), and splanchnic glucose production (SGP) were calculated as previously described (21) (Supplemental Figure 2). NSGB was negative in the fasting state; i.e., glucose concentrations were higher in the hepatic venous circulation (Supplemental Figure 3A). This did not differ between groups (Supplemental Table 1). On the other hand, during the clamp, mean NSGB became positive in response to the conditions present. At the end of the intermediate glucagon infusion (120 min), there were small differences in NSGB across the groups, though there were no significant differences between the 2 groups with obesity (Supplemental Table 1). No differences were apparent during the high glucagon infusion (240 min). SER, SGU, and SGP did not differ across the groups studied during fasting and during the clamp studies (Supplemental Table 1 and Supplemental Figure 3, B–D).

### Splanchnic leucine uptake, α-ketoisocaproic acid release, and leucine reamination and breakdown during fasting and then during the 2 stages of the hyperglycemic clamp.

**Figure 3:**
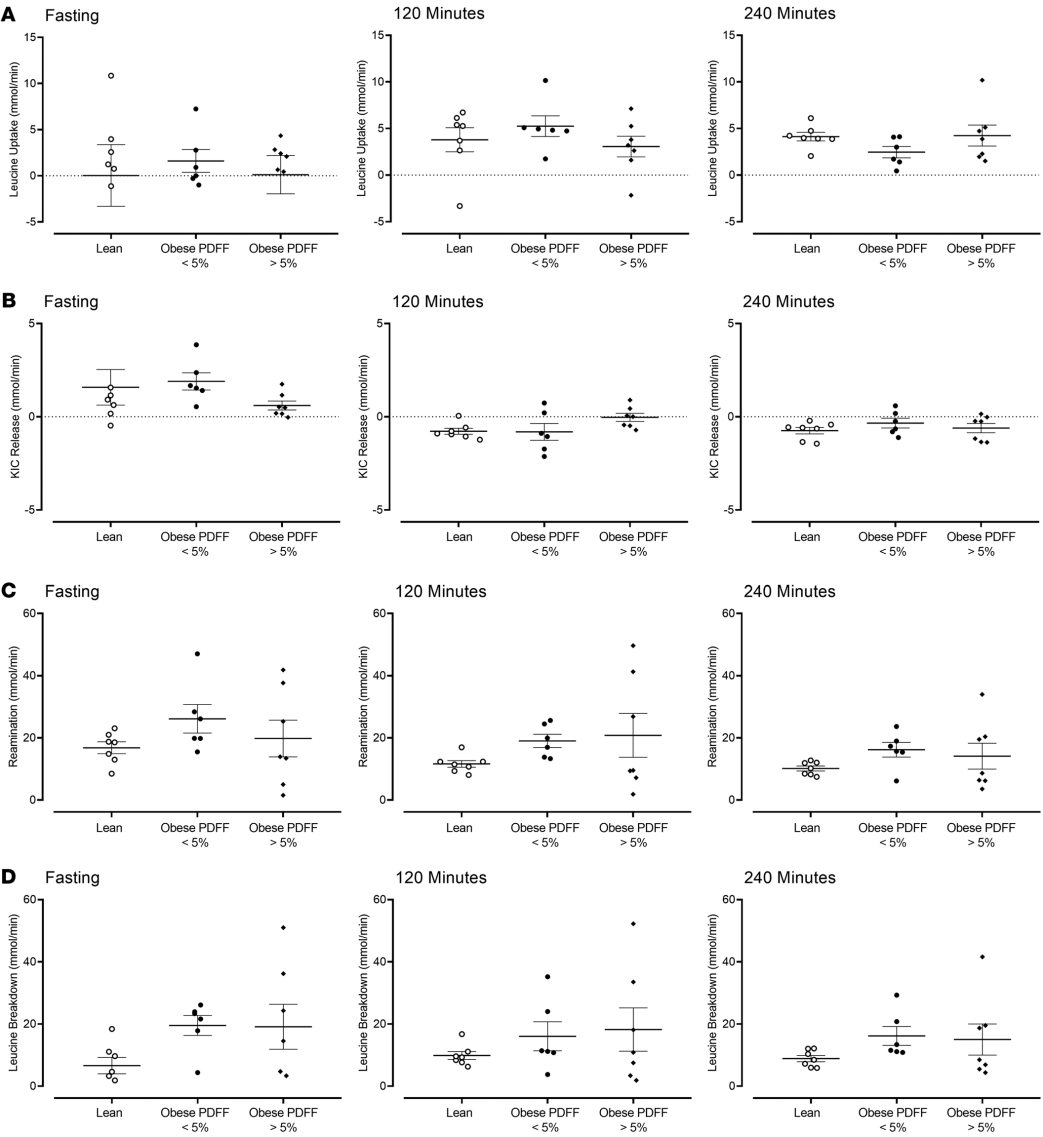
Leucine metabolism during the experiment. The mean (± SEM) together with the individual values of leucine uptake (**A**), KIC release (**B**), leucine reamination (**C**), and leucine breakdown (**D**) in lean patients (○), obese patients with a PDFF < 5% (●), and obese patients with a PDFF > 5% (♦). KIC, α-ketoisocaproic acid; PDFF, proton density fat fraction. *n* = 7 in the lean group, *n* = 6 in the obese group with PDFF < 5%, and *n* = 7 in the obese group with PDFF > 5%.

Leucine uptake (Figure 3A) by the splanchnic tissues was calculated (Supplemental Figure 4). During fasting it was unaffected by obesity with or without increased hepatic fat. In response to insulin and glucagon, leucine uptake increased but did not differ significantly across the groups during both rates of glucagon infusion. When the correlation of leucine uptake with hepatic fat as a continuous variable was examined, no relationship was observed (Supplemental Figure 5, A–C).

During fasting, α-ketoisocaproic acid (KIC) concentrations were higher in the arterial circulation compared with the hepatic vein; i.e., KIC was extracted by the liver (22) (Supplemental Figure 4). There was no statistically significant difference across groups (1.6 ± 0.9 vs. 1.9 ± 0.5 vs. 0.6 ± 0.2 mmol/min, *P* = 0.08 — Figure 3B). In response to insulin and glucagon, KIC was released by the liver, but no statistically significant across-group differences were apparent during intermediate glucagon infusion (–0.8 ± 0.2 vs. –0.8 ± 0.5 vs. –0.1 ± 0.2 mmol/min, *P* = 0.06 — Figure 3B). During high glucagon infusion there were no significant across-group differences (*P* = 0.61). However, when the correlation of leucine uptake with hepatic fat as a continuous variable was examined, a relationship was observed during intermediate glucagon infusion (Supplemental Figure 5, D–F). Note that KIC release increased as hepatic fat increased.

In the model previously described by Cheng et al. (22), reamination of KIC to leucine during steady-state conditions is equal to (therefore serving as a surrogate for) loss of ^15^N from l-[1-^13^C,^15^N]-leucine during conversion to KIC (Supplemental Figure 4). The rate of reamination did not differ across groups during fasting (17 ± 2 vs. 26 ± 5 vs. 20 ± 6 mmol/min, *P* = 0.22 — Figure 3C). In response to insulin and glucagon, there was a tendency toward higher reamination rates in the groups with obesity (independent of hepatic fat), but this was not significant during intermediate (12 ± 1 vs. 19 ± 2 vs. 22 ± 7 mmol/min, *P* = 0.15) and high glucagon infusion (10 ± 1 vs. 16 ± 2 vs. 14 ± 4 mmol/min, *P* = 0.39 — Figure 3C). Again, when the correlation of reamination with hepatic fat as a continuous variable was examined, a relationship was observed during intermediate glucagon infusion (Supplemental Figure 5, G–I). Reamination increased as hepatic fat increased.

Net leucine breakdown across the splanchnic tissues (Supplemental Figure 4) did not differ significantly across groups during fasting (7 ± 3 vs. 20 ± 3 vs. 19 ± 7 mmol/min, *P* = 0.09 — Figure 3D). During the subsequent 2 stages of the clamp, no significant differences were observed across the 3 groups (Figure 3D). There was a weak positive correlation of leucine breakdown with hepatic fat at all stages of the experiment (Supplemental Figure 5, J–L).

### Splanchnic balance of essential AAs during fasting and then during the 2 stages of the hyperglycemic clamp.

**Figure 4:**
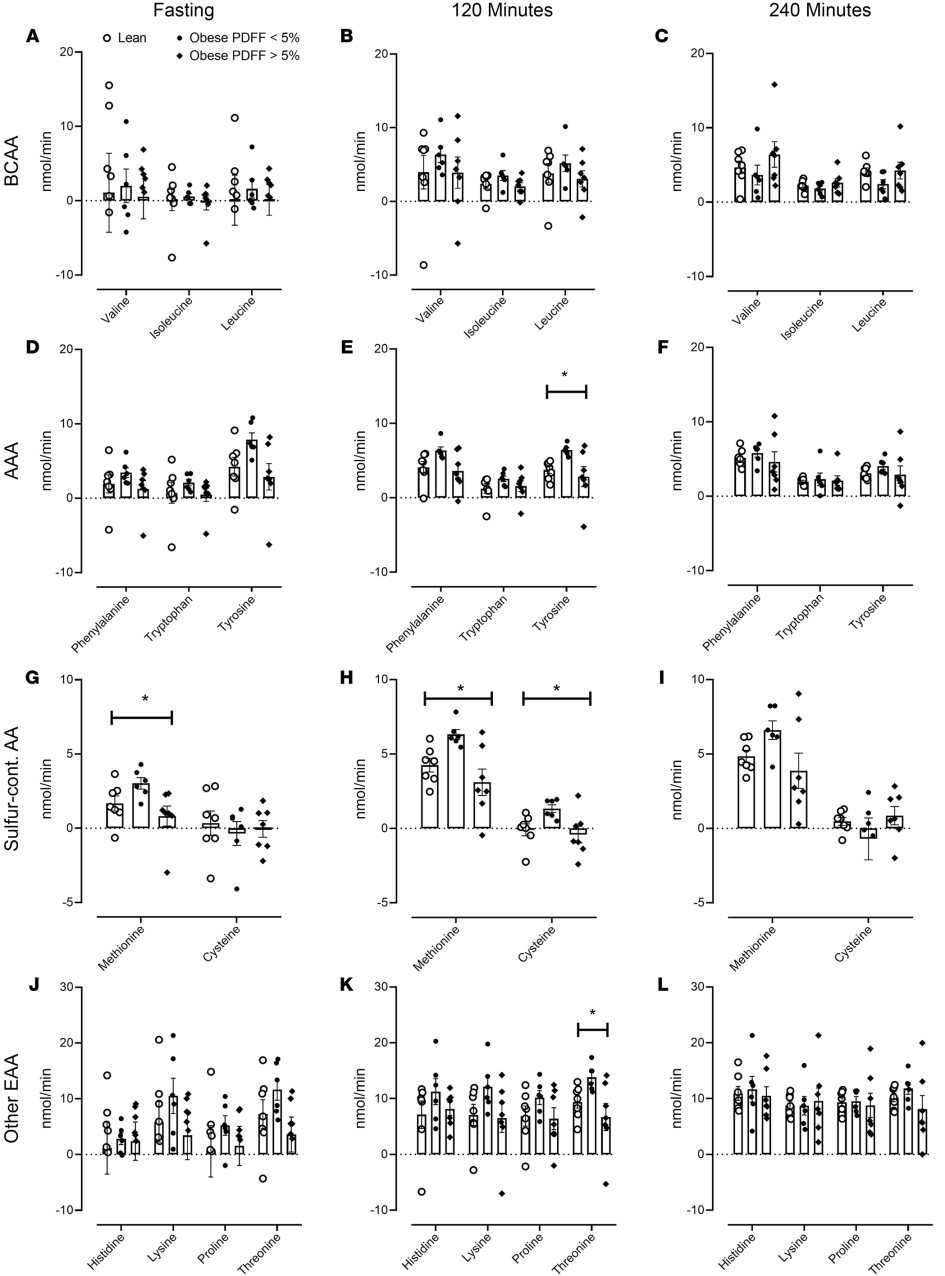
Splanchnic balance of essential amino acids during the experiment. The mean (± SEM) together with the individual values of splanchnic balance for branched-chain amino acids (BCAA), aromatic amino acids (AAA), sulfur-containing amino acids (Sulfur-cont. AA), and other essential amino acids (Other EAA), measured in lean patients (○), obese patients with a PDFF < 5% (●), and obese patients with a PDFF > 5% (♦), during fasting (**A**, **D**, **G**, and **J**), during intermediate glucagon infusion rates (120 minutes — **B**, **E**, **H**, and **K**), and during high glucagon infusion rates (240 minutes — **C**, **F**, **I**, and **L**). PDFF, proton density fat fraction. *P < 0.05 for a 1-way ANOVA test. *n* = 7 in the lean group, *n* = 6 in the obese group with PDFF < 5%, and *n* = 7 in the obese group with PDFF > 5%.

The splanchnic balance for individual essential AAs was calculated (Supplemental Figure 6). Positive values imply higher concentrations in the arterial circulation, i.e., hepatic uptake, and negative values imply the opposite, i.e., hepatic release. No significant across-group differences were apparent in the BCAAs (Figure 4, A–C).

In the case of aromatic amino acid (AAA; Figure 4, D–F), differences in tyrosine balance were apparent during intermediate glucagon infusion but not at other stages of the experiment. No correlation with hepatic fat content was noted (Supplemental Table 2).

The splanchnic balance of sulfur-containing AA (Figure 4, G–I) differed significantly during fasting (methionine only) and during intermediate, but not high, glucagon infusion. No correlation with hepatic fat content was noted (Supplemental Table 2).

Of the other essential AAs (Figure 4, J–L), the splanchnic balance of threonine differed during intermediate glucagon infusion, but no correlation with hepatic fat content was noted (Supplemental Table 2).

### Splanchnic balance of nonessential AAs during fasting and then during the 2 stages of the hyperglycemic clamp.

**Figure 5:**
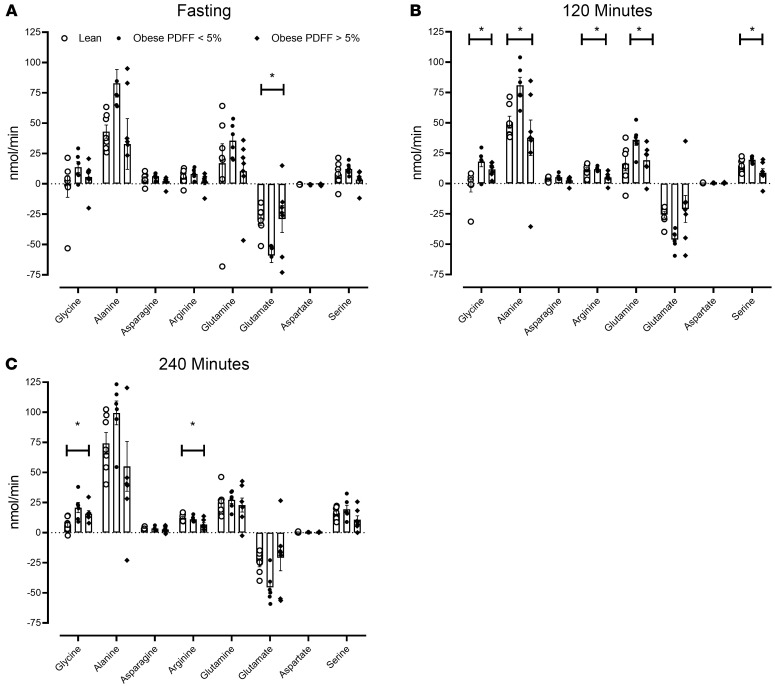
Splanchnic balance of nonessential AAs during the experiment. The mean (± SEM) together with the individual values of splanchnic balance for nonessential AAs during fasting (**A**), during intermediate glucagon infusion rates (120 minutes — **B**), and during high glucagon infusion rates (240 minutes — **C**) in lean patients (○), obese patients with a PDFF < 5% (●), and obese patients with a PDFF > 5% (♦). PDFF, proton density fat fraction. *P < 0.05 for a 1-way ANOVA test. *n* = 7 in the lean group, *n* = 6 in the obese group with PDFF < 5%, and *n* = 7 in the obese group with PDFF > 5%.

During the fasting state (Figure 5A) the splanchnic balance of glutamate differed significantly across groups (*P* = 0.03). However, these differences were no longer significant during both rates of glucagon infusion (Figure 5, B and C, respectively). The baseline splanchnic balance of glutamate correlated with weight but not hepatic fat (Supplemental Table 2).

Differences in the splanchnic balance of glycine and arginine became apparent during the experiment and (unlike those for alanine, glutamine, and serine) persisted to the end (Figure 5, B and C). Of these, only the splanchnic balance of arginine correlated with PDFF but not with weight (Supplemental Table 2).

### Splanchnic balance of AA metabolites during fasting and then during the 2 stages of the hyperglycemic clamp.

**Figure 6:**
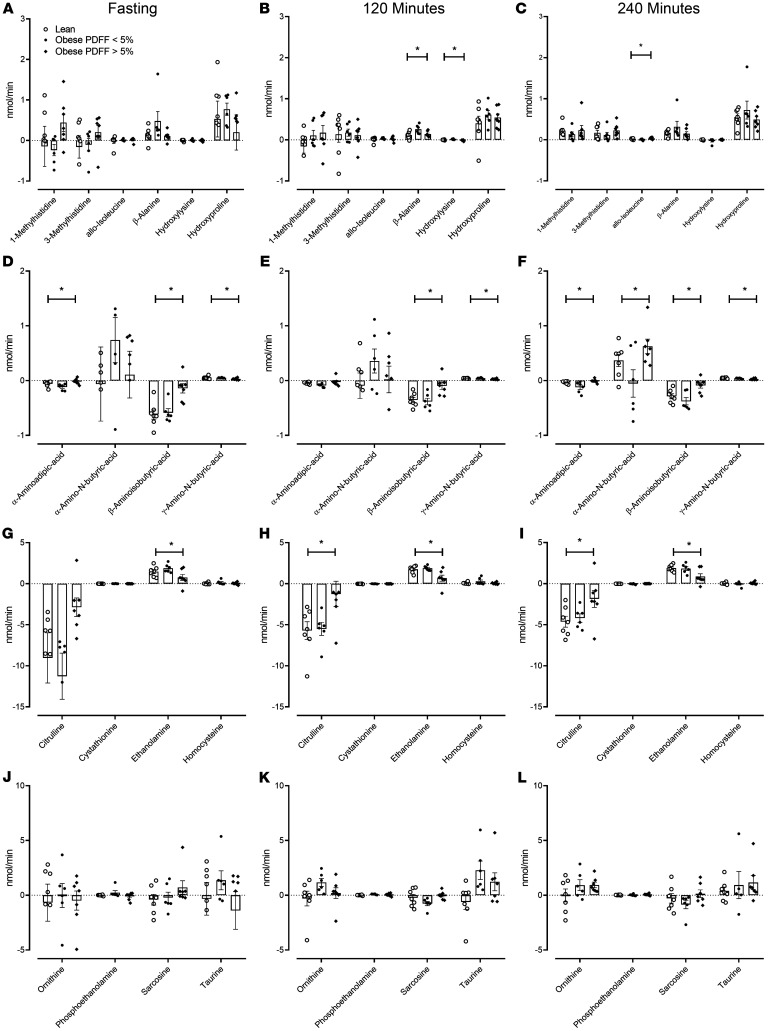
Splanchnic balance of AA metabolites during the experiment. The mean (± SEM) together with the individual values of splanchnic balance for metabolites measured during fasting (**A**, **D**, **G**, and **J**) and during intermediate (120 minutes — **B**, **E**, **H**, and **K**) and high glucagon infusion rates (240 minutes — **C**, **F**, **I**, and **L**) in lean patients (○), obese patients with a PDFF < 5% (●), and obese patients with a PDFF > 5% (♦). PDFF, proton density fat fraction. *P < 0.05 for a 1-way ANOVA test. *n* = 7 in the lean group, *n* = 6 in the obese group with PDFF < 5%, and *n* = 7 in the obese group with PDFF > 5%.

The splanchnic balance of several metabolites did not differ significantly during the fasting state (Figure 6A), but differences became apparent during the experiment (Figure 6, B and C). Of these, splanchnic balance of β-alanine correlated with weight but not PDFF (Supplemental Table 3). The splanchnic balance of allo-isoleucine and hydroxylysine correlated with PDFF, but this was inconsistent throughout the experiment (Supplemental Table 3).

α-Aminoadipic acid, α-amino-N-butyric acid, β-amino-isobutyric acid, and γ-amino-N-butyric acid all exhibited differences in splanchnic balance, though this was only consistent in the case of the latter 2 (Figure 6, D–F). β-Amino-isobutyric acid and γ-amino-N-butyric acid correlated with PDFF but not with weight (Supplemental Table 3).

Differences in the splanchnic balance of citrulline (Figure 6G) became significant during the experiment, when clamp conditions decreased net hepatic release (Figure 6, H and I). These differences correlated with PDFF (Supplemental Table 3). The differences for ethanolamine were less consistent over the duration of the experiment (Figure 6H and Supplemental Table 3).

Of the remaining metabolites analyzed (Figure 6, J–L), no differences in splanchnic balance were noted.

### SER of selected AAs and metabolites during fasting and then during the 2 stages of the hyperglycemic clamp.

The SER (Supplemental Figure 7) was calculated for AA and metabolites whose splanchnic balance correlated with weight (glutamic acid, glutamine, glycine, and β-alanine) and with PDFF (arginine, β-amino-isobutyric acid, citrulline, ethanolamine [not shown], and γ-amino-N-butyric acid — Supplemental Figure 8).

SER (which unlike splanchnic balance is independent of splanchnic blood flow) did not differ consistently across groups, but the differences, if any, were often driven by differences present at baseline. The change from baseline in response to insulin and glucagon did not differ across groups.

## Discussion

Glucagon secretion that is inappropriate for the prevailing glucose concentrations is increasingly recognized early in prediabetes (7). AAs are potent glucagon secretagogues. In turn, glucagon stimulates the catabolism of AA by the liver (16). The observation that prediabetes and T2DM are associated with increased circulating concentrations of some AAs (as well as relative or absolute hyperglucagonemia) has led to the hypothesis that impaired glucagon action on hepatic AA catabolism results in hyperglucagonemia and its attendant adverse effects on glucose metabolism (23). This hypothesis has been bolstered by the observation that hyperglucagonemia and higher circulating AA are present in MASLD (24). In accordance with this hypothesis, Suppli et al. reported that hepatic steatosis impaired ureagenesis and AA catabolism in response to glucagon (14).

Glucagon signals through its 7-transmembrane helix receptor, activating G_αs_-coupled proteins that increase cAMP and activate cAMP response element binding protein and PKA, which act in concert to decrease glycolysis and increase gluconeogenesis and glycogenolysis (25). Glucagon receptor activation also stimulates intrahepatic lipolysis (26). However, although hepatic steatosis has been proposed as a surrogate for hepatic glucagon resistance with a bifurcation in hepatic glucagon action on lipids and carbohydrates in rodents (13), this has not been observed in humans to date (27). Indeed, several mechanisms for selective resistance of AA metabolism to glucagon have been proposed (28), but these have not been directly tested in humans.

We sought to address this question using an experimental design (Supplemental Figure 1) that enabled direct measurement of leucine oxidation across the splanchnic bed. In addition, the measurement of arterial and hepatic vein concentrations of all other AAs enabled assessment of their handling by the splanchnic tissues in experimental conditions intended to mimic postcibal glucose, AA, insulin, and glucagon concentrations. More importantly, we designed an experiment to overcome the technical limitations of prior work (14) while approximating postprandial conditions.

The first priority was to ensure that we could differentiate the effects of obesity from those of MASLD. To do so, we studied lean patients without any evidence of hepatic steatosis together with patients with obesity with and without hepatic steatosis. Lower insulin infusion rates resulting in concentrations similar to those encountered during fasting may exacerbate the effects of glucagon on metabolism (14). However, the conclusions would have little relevance to normal postprandial physiology, especially in patients with MASLD, where insulin rises significantly in response to food ingestion. Therefore, we infused insulin at rates that resulted in peripheral concentrations similar to those observed in the postprandial period. This enabled us to maintain a hyperglycemic clamp and ensure that glucose concentrations did not differ across the groups studied, as was the case with lower insulin concentrations (14).

At the time of screening, an oral glucose tolerance test (OGTT) was used to measure β cell function and insulin action. Although no differences in these parameters between the obese patients with and without hepatic steatosis were noted (Table 1), during the experiment it was apparent that peripheral glucose disposal was further impaired in the group with a PDFF > 5%, compared with the group with obesity and a PDFF < 5% (Figure 2, A and B). On the other hand, hepatic responses to the experimental conditions did not differ across all groups (Figure 2C, Supplemental Figures 2 and 3, and Supplemental Table 1). This implies that hepatic carbohydrate metabolic responses to rising glucagon are preserved in the presence of hepatic steatosis.

We used doubly labeled leucine and the model previously described by Cheng et al. (22) to estimate leucine uptake, KIC release, leucine reamination, and leucine breakdown (Figure 3 and Supplemental Figure 4). As before (29), leucine reamination was used as a surrogate for the rate of transamination (loss of ^15^N) and conversion to KIC, enabling measurement of the first step of leucine catabolism in response to glucagon (30, 31). This did not differ across groups (Figure 3). To ensure that we did not miss an effect of hepatic fat with glucagon responses, we examined the relationship of these fluxes with hepatic fat (Supplemental Figure 5). If anything, the (positive) correlations observed would suggest an enhanced response to glucagon in the presence of increased hepatic fat. This would tend to refute the hypothesis that hepatic fat is a marker of hepatic glucagon insensitivity. It could also suggest that decreased insulin action in people with MASLD “permits” greater effects of glucagon on leucine metabolism at a given insulin concentration. This is certainly true of glucose metabolism with overt steatohepatitis associated with metabolic dysfunction (32), but whether it applies to AA metabolism will require further study.

We subsequently used splanchnic balance (Supplemental Figure 6) to screen for differences in metabolism of other AAs. Some across-group differences were identified, though these differences mostly correlated with weight and not hepatic fat. This was not the case for arginine, where splanchnic balance correlated with hepatic fat (Supplemental Table 2). However, the changes in SER (which are independent from alterations in blood flow) from baseline in response to rising insulin and glucagon (120 min) and further increases in glucagon (240 min) followed the same pattern as in lean patients (Supplemental Figure 8) and did not differ across groups.

In addition to AA, we measured the splanchnic balance of metabolites (some AA-derived) that are part of our standard AA panel (see Methods). We again used splanchnic balance (Supplemental Figure 6) to screen for differences in their metabolism (Figure 6). The splanchnic balance of β-aminoisobutyric acid and γ-amino-N-butyric acid was associated with PDFF rather than weight (Supplemental Table 3). This reflected significant differences in their arterial and hepatic venous concentrations in obese patients with a PDFF > 5%, but the change (or lack thereof) in SER from fasting during hyperinsulinemia and hyperglucagonemia did not differ from that in the other groups (Supplemental Figure 8). Similar patterns were observed for citrulline and ethanolamine (not shown).

Coincidentally a metabolite whose splanchnic balance differed across groups was α-aminoadipic acid, which is associated with increased conversion of prediabetes to T2DM (9). This seems to be released by the liver to a greater extent in obesity. β-Aminoisobutyric acid (thought to improve insulin action) (33) also seems to be released by the liver but at lower rates in people with obesity and hepatic steatosis (Supplemental Figure 6F). SER does not change appreciably from baseline during the experiment (as is the case for lean patients and people with obesity and a PDFF < 5%). γ-Amino-N-butyric acid (a compound with unclear metabolic effects) is extracted by the splanchnic bed to a lesser extent in people with obesity and hepatic steatosis (Supplemental Figure 8H). Whether these metabolites may serve as useful markers of hepatic steatosis — and the metabolic abnormalities associated with this phenotype — remain to be ascertained.

The experiment has significant strengths, including the placement of arterial and hepatic venous catheters allowing the comprehensive measurement of AAs and other metabolites of interest across the liver and the tracer-based measurement of hepatic metabolism. This together with the tests at screening allowed detailed phenotypic characterization of the patients, as well as recruitment of obese patients, otherwise matched for metabolic and anthropometric characteristics, with or without hepatic steatosis. Fulfilling this requirement was necessary to enable us to address our primary hypothesis and differentiate the effect of obesity from that of hepatic steatosis — a limitation of prior studies (14). Although a PDFF > 5% has consistently been used as a marker for significant hepatic steatosis (34), it is possible that metabolic effects could be present at lower values. To overcome this, we examined the correlation between various endpoints and PDFF in a continuous fashion. This strengthened our conclusions.

As with all experiments, there are some limitations that need to be considered when interpreting the results of the experiment. The first is the relatively small sample size studied, a consequence of the complexity, expense, and invasiveness of the experimental design. On the other hand, the ability to measure the extraction of multiple AAs across the liver coupled with state-of-the-art techniques makes it unlikely that we failed to detect a physiologically significant defect in hepatic glucagon action. This is borne out by the overlapping distribution of various endpoints across all 3 groups studied and by the post hoc correlation with PDFF. Another consequence of the small sample size is the unbalanced sex distribution. Although we have not previously studied the response of AA metabolism to glucagon, we have not observed an effect of sex on responses of carbohydrate metabolism to glucagon (35).

Despite our efforts to suppress endogenous insulin secretion using somatostatin, it is clear that some portal insulin secretion persisted in obese patients and may have attenuated some of glucagon’s effects on hepatic metabolism. This is unlikely, given the minor differences in C-peptide concentrations (especially in the setting of physiological hyperinsulinemia) and the degree of impaired insulin action present in the affected patients. Finally, the constraints of the experimental design necessitated that macronutrients were delivered into the peripheral rather than the portal circulation. Due to hepatic zonation, it is possible that we missed effects due to zone-specific gradients in hormonal and nutrient exposure, although this is unlikely given the duration of each stage of the experiment (36), allowing time for equilibration. In addition, in a prior human experiment after portal delivery of AA, equilibration of hepatic vein and portal vein concentrations occurred rapidly (37). From this series of experiments in humans, where we sought to mimic postprandial conditions with simultaneous (physiologic) hyperinsulinemia, hyperglycemia, and hyperglucagonemia, we conclude that there is no evidence of resistance to the actions of glucagon on AA metabolism conferred by hepatic steatosis. Abnormalities of glucagon secretion have been associated with impaired insulin action and with obesity (38, 39) but based on current evidence are unlikely to be explained by selective hepatic glucagon resistance.

## Methods

### Sex as a biological variable.

We studied male and female patients. We did not report findings separately.

### Screening.

After approval from the Mayo Clinic Institutional Review Board, we recruited patients using intramural and extramural advertising. To enhance our ability to recruit patients with hepatic steatosis, we wrote to patients previously identified in the Mayo Clinic Biobank (40, 41) as having hepatic steatosis. We also used the fatty liver index to identify patients outside of the biobank at increased risk of hepatic steatosis (42). Eligible patients had no history of chronic illness (including diabetes), macro- or microvascular disease, or upper gastrointestinal surgery. Additionally, they were not taking medications that could affect weight or glucose metabolism. The alcohol use disorders identification test questionnaire (43) was used to screen for alcohol excess.

Potentially eligible patients interested in participating were invited to the Clinical Research and Trials Unit (CRTU) for a screening visit. After written, informed consent was obtained, participants underwent a 2-hour, 7-sample (0, 10, 20, 30, 60, 90, and 120 min), 75 g OGTT. This allows estimation of insulin secretion and action using the oral minimal model and classification of patients’ glucose tolerance status as previously described (44). All patients were instructed to follow a weight-maintenance diet containing 55% carbohydrate, 30% fat, and 15% protein for at least 3 days prior to the study. Body composition was measured at the time of screening using dual-energy X-ray absorptiometry (iDXA scanner; GE). Liver fat was measured by MRI using PDFF (45), where a value ≥5% implies ≥grade 1 steatosis. Liver stiffness was measured by MRI elastography as previously described (46, 47), where a value <2.5 kPa is considered normal and a value in the range of 3.0 to 3.5 kPa suggests stage 1–2 fibrosis.

### Experimental design.

See Supplemental Figure 1. Participants were admitted to the CRTU at 1700 on the day before the study. After consuming a standard 10 kcal/kg caffeine-free meal, they fasted overnight other than sips of water when thirsty. The following morning at 0700 (–210 min), 2 forearm vein catheters were placed to allow for nutrient and hormone infusions. A urinary catheter was also placed at this time. Prior to their departure from the CRTU to the interventional radiology suite at 0730 (–180 min), a primed (10 μCi prime, 0.1 μCi/min continuous) infusion containing trace amounts of glucose labeled with [3-^3^H] glucose was started and continued at this rate till 1030 (0 min). In the interventional radiology suite a hepatic vein catheter was placed via the femoral vein under fluoroscopic guidance (21). A femoral artery catheter was also placed. Infusion of indocyanine green (0.25 mg/min) and l-[1-^13^C,^15^N]-leucine (7.5 μmol/kg prime, 7.5 μmol/kg/h continuous) was also started at this time. [U-^13^C]-palmitate (300 nmol/min) commenced at 0830 (–120 min) after their return from interventional radiology and continued till the end of the study.

At 1030 (0 min) another glucose infusion, also labeled with [3-^3^H] glucose, commenced, and the infusion rate varied to produce peripheral glucose concentrations of ~160 mg/dL (9 mmol/L). The infusion rate containing trace amounts of glucose labeled with [3-^3^H] glucose decreased (0.03 μCi/min) to minimize anticipated increases in specific activity caused by suppression of EGP (48). To mimic ingestion of a protein load, Clinisol (15%, 0.003 mL/kg/min; 51% essential AA, 18% BCAA, 9% AAA; Baxter Healthcare) was also infused at this time. In addition, insulin (0.8 mU/kg/min), glucagon (1.5 ng/kg/min — intermediate glucagon infusion rate), and somatostatin (60 ng/kg/min) were infused. The insulin infusion was kept constant for the remainder of the study, but at 1230 (120 min) the glucagon infusion rate doubled to 3.0 ng/kg/min (high glucagon infusion rate). Blood samples from the femoral vein, hepatic vein, and femoral artery were obtained at –30 to 0 (baseline), 90 to 120 (moderate glucagon), and 210 to 240 minutes (high glucagon). At the end of the study (1430 — 240 min), all vascular catheters were removed. Site care was performed as per institutional guidelines with the application of localized pressure at the sites of femoral vascular access. Participants consumed a late lunch but remained supine in bed until 1730 when they were mobilized. In the absence of site concerns, patients left the CRTU once they were felt to be safe to do so.

### Analytic techniques.

All blood was immediately placed on ice after collection, centrifuged at 4^o^C, separated, and stored at –80°C until assay. Plasma glucose concentrations were measured using a Yellow Springs glucose analyzer. Glucagon was measured using an ELISA (Mercodia; ref. 49). C-peptide was measured using EMD Millipore reagents. Insulin was measured using a chemiluminescence assay with reagents obtained from Beckman (Access Assay). [3-^3^H] glucose–specific activity was measured by liquid scintillation counting following deproteinization (50). Indocyanine green concentrations in the serum were measured using a Spectramax M2 spectrophotometer (Molecular Devices) at the 805 nm wavelength (51). AAs and their metabolite data were acquired on the Thermo Fisher Scientific TSQ Quantiva mass spectrometer coupled with a Waters Acquity UPLC system as previously described (52).

### Calculations.

EGP and glucose disposal (Rd) were calculated as before (48, 53). *S_i_* and β cell responsivity (Φ) were calculated (54) from the plasma glucose, insulin, and C-peptide concentrations during the screening OGTT (55).

The mean values of isotope enrichment during fasting (–30 to 0 min), during intermediate glucagon infusion (90 to 120 min), and during high glucagon infusion (210 to 240 min) were used for all tracer-based calculations of AA kinetics. The calculation of leucine carbon and nitrogen flux and KIC reamination to leucine under steady-state conditions (29) utilized the model previously described by Cheng et al. (22).

Splanchnic plasma flow was calculated by dividing the indocyanine green infusion rate by the arterial-hepatic venous concentration gradient of the dye. Where necessary, dividing the plasma flow by 1-hematocrit provided splanchnic blood flow (51). The splanchnic balance for a given AA was calculated from the arteriovenous difference of AA concentration across the liver, multiplied by splanchnic blood flow, while the SER was calculated from the arteriovenous difference of AA concentration across the liver, divided by the arterial concentration (Supplemental Figure 6 for splanchnic balance and Supplemental Figure 7 for SER).

### Statistics.

All continuous data are summarized as means ± SEM. AUC and area above basal were calculated using the trapezoidal rule. One-way ANOVA and a Tukey’s post hoc test were used to determine between-group differences (parametric data). A Kruskal-Wallis test followed by Dunn’s post hoc test was used for nonparametric data. When necessary, linear regression was performed using BlueSky Statistics software v. 7.10 and Prism 5 (GraphPad Software). A *P* < 0.05 was considered statistically significant. Although no data existed for our experimental conditions, Nygren et al. observed (mean ± SD) leucine reamination rate of 66.8 ± 9.5 mmol/min (29). Assuming a similar variability, 6 patients per group would give us the ability to detect (80% power, α = 0.05) a 20% difference in reamination rate attributable to hepatic steatosis.

### Study approval.

The Mayo Clinic Institutional Review Board approved the study and associated study documents. It was subsequently registered at ClinicalTrials.gov. Somatostatin was infused under an IND approved by the FDA.

### Data availability.

All data reported in this paper are provided in an accompanying Supporting Data Values file available for download. This paper does not report original code. Any additional information required to reanalyze the data reported in this paper is available from the lead contact upon request.

## Author contributions

HEC, SM, and AME researched data, ran the studies, contributed to the discussion, and reviewed/edited manuscript; FB and CDM supervised the mathematical modeling, contributed to the discussion, and reviewed/edited manuscript; SMT, MJ, CJF, and JCA placed the catheters necessary to complete the study, contributed to the discussion, and reviewed/edited manuscript; KRB supervised the statistical analyses; MDJ and KSN contributed to the design and analysis of the study, contributed to the discussion, and reviewed/edited manuscript; AV designed the study, oversaw its conduct, researched data, and wrote the first draft of the manuscript. AV is the guarantor of this work and, as such, has full access to all the data in the study and takes responsibility for the integrity of the data and the accuracy of the data analysis.

## Funding support

This work is the result of NIH funding, in whole or in part, and is subject to the NIH Public Access Policy. Through acceptance of this federal funding, the NIH has been given a right to make the work publicly available in PubMed Central.

- NIH National Institute of Diabetes and Digestive and Kidney Diseases DK116231.
- AV by DK78646, DK116231, and DK126206.
- CDM, KRB, and MDJ by DK116231.

## Supplementary Material

Supplemental data

ICMJE disclosure forms

Supporting data values

## Figures and Tables

**Table 1:**
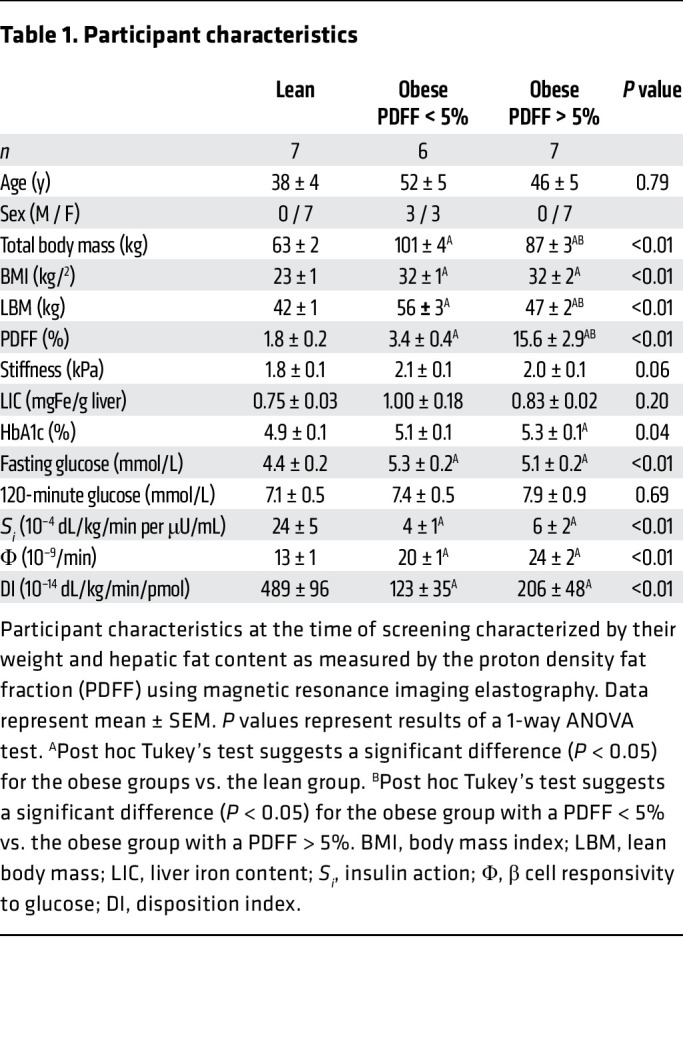
Participant characteristics

## Version 1 12/23/2025

In-Press Preview

## Version 2 03/02/2026

Electronic publication

## References

[1] Ogden CL (2016). Trends in obesity prevalence among children and adolescents in the United States, 1988-1994 through 2013-2014. JAMA.

[2] Younossi ZM (2016). Global epidemiology of nonalcoholic fatty liver disease-Meta-analytic assessment of prevalence, incidence, and outcomes. Hepatology.

[3] Franch-Nadal J (2018). Fatty liver index is a predictor of incident diabetes in patients with prediabetes: The PREDAPS study. PLoS One.

[4] Balkau B (2010). Nine-year incident diabetes is predicted by fatty liver indices: the French D.E.S.I.R. study. BMC Gastroenterol.

[5] Zelber-Sagi S (2013). Non-alcoholic fatty liver disease independently predicts prediabetes during a 7-year prospective follow-up. Liver Int.

[6] Albright AL, Gregg EW (2013). Preventing type 2 diabetes in communities across the U.S.: the National Diabetes Prevention Program. Am J Prev Med.

[7] Mohan S (2025). Abnormal glucagon secretion contributes to a longitudinal decline in glucose tolerance. J Clin Endocrinol Metab.

[8] Wang TJ (2011). Metabolite profiles and the risk of developing diabetes. Nat Med.

[9] Wang TJ (2013). 2-Aminoadipic acid is a biomarker for diabetes risk. J Clin Invest.

[10] Dean ED (2017). Interrupted glucagon signaling reveals hepatic α cell axis and role for L-glutamine in α cell proliferation. Cell Metab.

[11] Shah P (2000). Lack of suppression of glucagon contributes to postprandial hyperglycemia in subjects with type 2 diabetes mellitus. J Clin Endocrinol Metab.

[12] Baum JI (2009). Glucagon acts in a dominant manner to repress insulin-induced mammalian target of rapamycin complex 1 signaling in perfused rat liver. Am J Physiol Endocrinol Metab.

[13] Li S (2010). Bifurcation of insulin signaling pathway in rat liver: mTORC1 required for stimulation of lipogenesis, but not inhibition of gluconeogenesis. Proc Natl Acad Sci U S A.

[14] Suppli MP (2020). Glucagon resistance at the level of amino acid turnover in obese subjects with hepatic steatosis. Diabetes.

[15] Winther-Sorensen M (2020). Glucagon acutely regulates hepatic amino acid catabolism and the effect may be disturbed by steatosis. Mol Metab.

[16] Dean ED (2020). A primary role for α-cells as amino acid sensors. Diabetes.

[17] Bock G (2007). Effects of nonglucose nutrients on insulin secretion and action in people with pre-diabetes. Diabetes.

[18] Robertson RP (2015). Assessment of β-cell mass and α- and β-cell survival and function by arginine stimulation in human autologous islet recipients. Diabetes.

[19] Zeini M (2024). The longitudinal effect of diabetes-associated variation in TCF7L2 on islet function in humans. Diabetes.

[20] Bock G (2006). Pathogenesis of pre-diabetes: mechanisms of fasting and postprandial hyperglycemia in people with impaired fasting glucose and/or impaired glucose tolerance. Diabetes.

[21] Basu A (2001). Type 2 diabetes impairs splanchnic uptake of glucose but does not alter intestinal glucose absorption during enteral glucose feeding: additional evidence for a defect in hepatic glucokinase activity. Diabetes.

[22] Cheng KN (1985). Direct determination of leucine metabolism and protein breakdown in humans using L-[1-13C, 15N]-leucine and the forearm model. Eur J Clin Invest.

[23] Wewer Albrechtsen NJ (2019). The liver-α-cell axis and type 2 diabetes. Endocr Rev.

[24] Wewer Albrechtsen NJ (2018). Hyperglucagonemia correlates with plasma levels of non-branched-chain amino acids in patients with liver disease independent of type 2 diabetes. Am J Physiol Gastrointest Liver Physiol.

[25] Petersen MC (2017). Regulation of hepatic glucose metabolism in health and disease. Nat Rev Endocrinol.

[26] Perry RJ (2020). Glucagon stimulates gluconeogenesis by INSP3R1-mediated hepatic lipolysis. Nature.

[27] Ter Horst KW (2021). Hepatic insulin resistance is not pathway selective in humans with nonalcoholic fatty liver disease. Diabetes Care.

[28] Janah L (2019). Glucagon receptor signaling and glucagon resistance. Int J Mol Sci.

[29] Nygren J, Nair KS (2003). Differential regulation of protein dynamics in splanchnic and skeletal muscle beds by insulin and amino acids in healthy human subjects. Diabetes.

[30] Charlton MR (1996). Evidence for a catabolic role of glucagon during an amino acid load. J Clin Invest.

[31] Charlton MR, Nair KS (1998). Role of hyperglucagonemia in catabolism associated with type 1 diabetes: effects on leucine metabolism and the resting metabolic rate. Diabetes.

[32] Sabatini S (2024). Hepatic glucose production rises with the histological severity of metabolic dysfunction-associated steatohepatitis. Cell Rep Med.

[33] Tanianskii DA (2019). Beta-aminoisobutyric acid as a novel regulator of carbohydrate and lipid metabolism. Nutrients.

[34] Azizi N (2025). Evaluation of MRI proton density fat fraction in hepatic steatosis: a systematic review and meta-analysis. Eur Radiol.

[35] Adams JD (2021). Insulin secretion and action and the response of endogenous glucose production to a lack of glucagon suppression in nondiabetic subjects. Am J Physiol Endocrinol Metab.

[36] Hall Z (2017). Lipid zonation and phospholipid remodeling in nonalcoholic fatty liver disease. Hepatology.

[37] Yamakawa M (1997). Peptide digestion and absorption in humans: portal vein, hepatic vein, and peripheral venous amino acid concentrations. Asia Pac J Clin Nutr.

[38] Sharma A (2018). Impaired insulin action is associated with increased glucagon concentrations in nondiabetic humans. J Clin Endocrinol Metab.

[39] Kohlenberg JD (2023). Differential contribution of alpha and beta cell dysfunction to impaired fasting glucose and impaired glucose tolerance. Diabetologia.

[40] Allen AM (2018). Nonalcoholic fatty liver disease incidence and impact on metabolic burden and death: A 20 year-community study. Hepatology.

[41] Peeraphatdit TB (2020). A cohort study examining the interaction of alcohol consumption and obesity in hepatic steatosis and mortality. Mayo Clin Proc.

[42] Bedogni G (2006). The Fatty Liver Index: a simple and accurate predictor of hepatic steatosis in the general population. BMC Gastroenterol.

[43] Saunders JB (1993). Development of the alcohol use disorders identification test (AUDIT): WHO collaborative project on early detection of persons with harmful alcohol consumption--II. Addiction.

[44] Sathananthan A (2012). A concerted decline in insulin secretion and action occurs across the spectrum of fasting and postchallenge glucose concentrations. Clin Endocrinol (Oxf).

[45] Moura Cunha G (2021). Quantitative magnetic resonance imaging for chronic liver disease. Br J Radiol.

[46] Ozturk A (2022). Liver fibrosis assessment: MR and US elastography. Abdom Radiol (NY).

[47] Venkatesh SK (2013). Magnetic resonance elastography of liver: technique, analysis, and clinical applications. J Magn Reson Imaging.

[48] Vella A, Rizza RA (2009). Application of isotopic techniques using constant specific activity or enrichment to the study of carbohydrate metabolism. Diabetes.

[49] Lund A (2016). Evidence of extrapancreatic glucagon secretion in man. Diabetes.

[50] Basu R (2003). Use of a novel triple-tracer approach to assess postprandial glucose metabolism. Am J Physiol Endocrinol Metab.

[51] Basu A (2000). Effects of type 2 diabetes on the ability of insulin and glucose to regulate splanchnic and muscle glucose metabolism: evidence for a defect in hepatic glucokinase activity. Diabetes.

[52] Lanza IR (2010). Quantitative metabolomics by H-NMR and LC-MS/MS confirms altered metabolic pathways in diabetes. PLoS One.

[53] Smushkin G (2013). The effect of a bile acid sequestrant on glucose metabolism in subjects with type 2 diabetes. Diabetes.

[54] Cobelli C (2014). The oral minimal model method. Diabetes.

[55] Breda E (2001). Oral glucose tolerance test minimal model indexes of beta-cell function and insulin sensitivity. Diabetes.

