# Supplementary material for: Hepatic steatosis in humans is associated with preserved glucagon action on amino acid metabolism
Source: J Clin Invest. 2025 Dec 23;136(5):e200913. doi: 10.1172/JCI200913 (PMC12948432; doi:10.1172/JCI200913)
Supplement: Supplemental data [file jci-136-200913-s195.pdf]

Hepatic steatosis in humans is associated with preserved glucagon action on amino acid metabolism – Supplementary Data

Hannah E. Christie PhD[1], Sneha Mohan MBBS[1], Aoife M. Egan MB, BCh, PhD[1], Federica Boscolo[2], Chiara Dalla Man PhD[2], Scott M. Thompson MD PhD[3], Michael Jundt MD[3], Chad J. Fleming MD[3], James C. Andrews MD[3], Kent R. Bailey PhD[4], Michael D. Jensen MD[1], K. Sree Nair MD PhD[1], *Adrian Vella MD[1].

0000-0002-0606-6778 Hannah E. Christie
0000-0002-4840-1802 Sneha Mohan
0000-0003-0379-9279 Aoife M. Egan
0009-0003-0545-7288 Federica Boscolo
0000-0002-4908-0596 Chiara Dalla Man
0000-0001-5589-8389 Michael Jensen
0000-0001-6493-7837 Adrian Vella

[1]Division of Endocrinology, Diabetes & Metabolism, Mayo Clinic, Rochester, Minnesota, USA
[2]Department of Information Engineering, University of Padova, Padova, Italy
[3]Division of Vascular and Interventional Radiology, Mayo Clinic, Rochester, Minnesota, USA
[4]Division of Biomedical Statistics and Informatics, Mayo Clinic, Rochester, Minnesota, USA

Abbreviated title: Glucagon effects on hepatic metabolism

**Address for Correspondence/Reprints:*
Adrian Vella, MD
Endocrine Research Unit
Mayo Clinic College of Medicine
200 First ST SW, 5-194 Joseph
Rochester, MN 55905
(T) 507-255-6515
(F) 507-255-4828
Email: vella.adrian@mayo.edu

Clinical Trials.Gov: NCT05500586
IND (for Somatostatin): 116569

Conflict of Interest Statement

Dr. Vella and has consulted for Boehringer-Ingelheim, Neurotronics and Rezolute. None of the other authors declare conflict of interests related to this study.

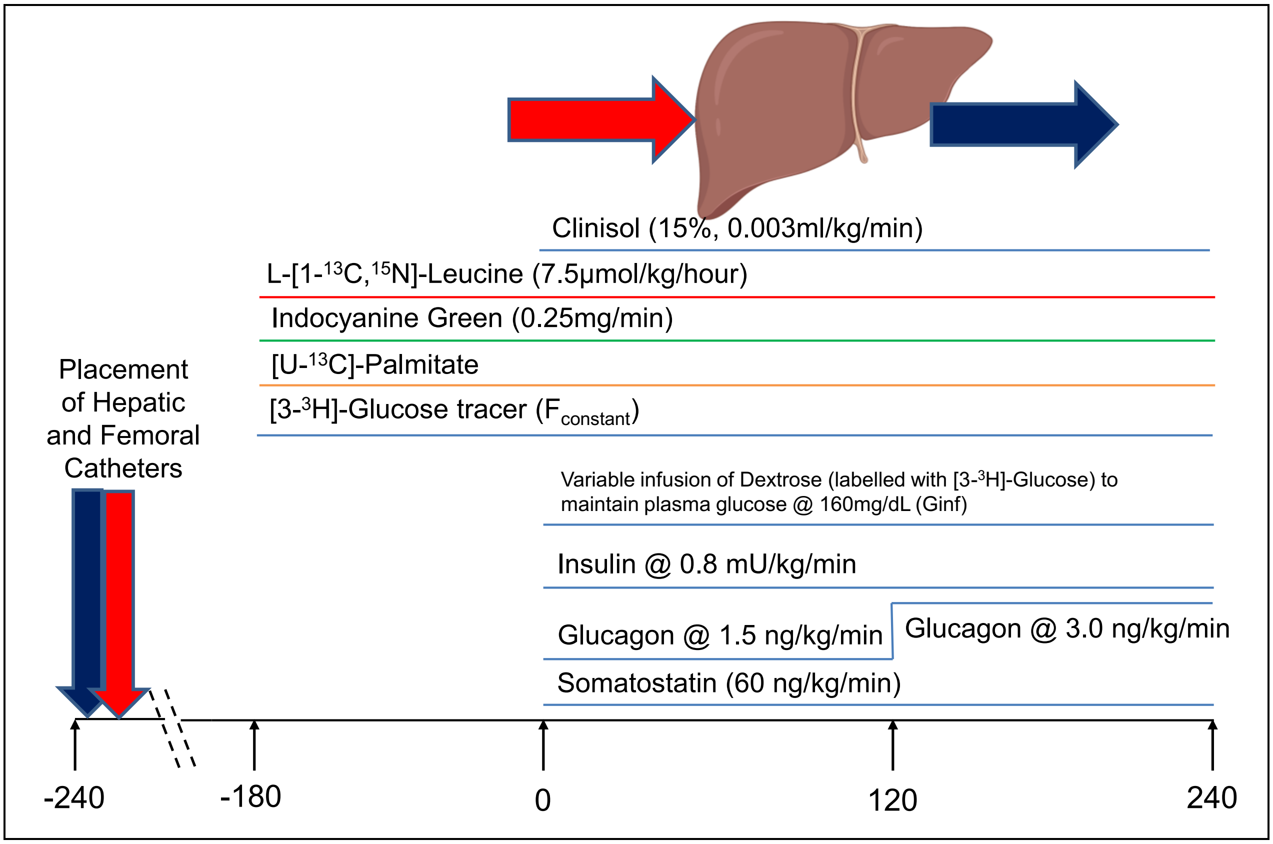

Supplementary Figure 1: Experimental Design

$$NSGB = \left(FA_{glucose} - HV_{glucose}\right) * Splanchnic\ Blood\ Flow$$

$$SER = \frac{[3 - {}^{3}H]\ glucoseFA - [3 - {}^{3}H]\ glucoseHV}{[3 - {}^{3}H]\ glucose_{FA}}$$

$$SGU = SER * SBF * [Glucose]$$

$$SGP = NSGB - SGU$$

Supplementary Figure 2: Equations used to calculate Net Splanchnic Glucose Balance (NSGB), Splanchnic Extraction Ratio (SER), Splanchnic Glucose Uptake (SGU) and Splanchnic Glucose Production during fasting and the two stages of the clamp.

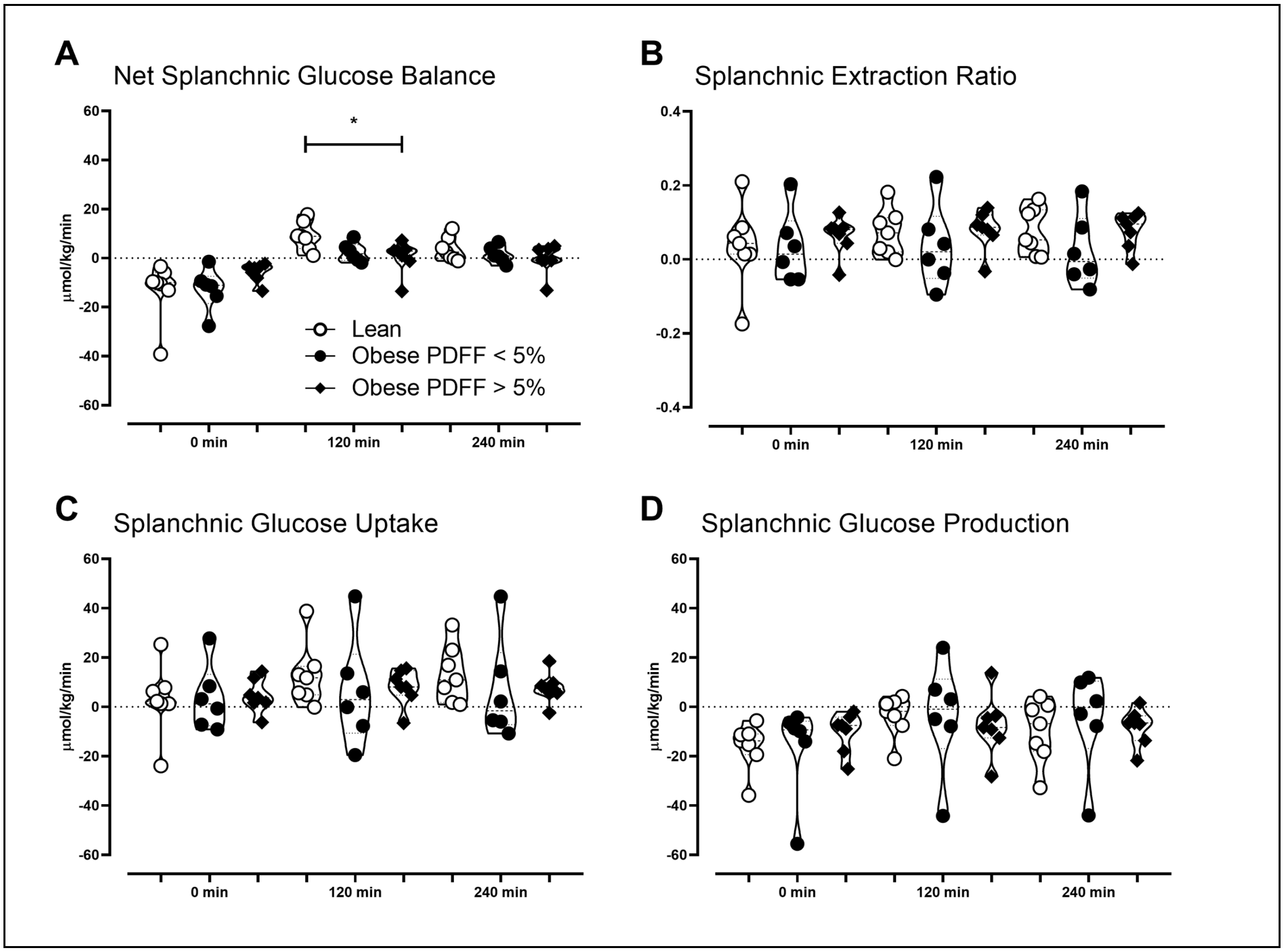

Supplementary Figure 3: Dot and violin plots show individual values for Net Splanchnic Glucose Balance (NSGB), Splanchnic Extraction Ratio (SER), Splanchnic Glucose Uptake (SGU) and Splanchnic Glucose Production during fasting (0 min) and the intermediate and high glucagon infusion rates (120 min and 240 min, respectively) of the clamp in lean subjects (○), obese subjects with a PDFF < 5% (●) and obese subjects with a PDFF > 5% (♦). * Represents a *P*-value < 0.05 for a one-way analysis of variance (ANOVA) test.

		Lean (n = 7)	Obese PDFF < 5% (n = 6)	Obese PDFF > 5% (n = 7)	*P*-value
**NSGB (µmol/kg/min)**	**Fasting**	-13.1 ± 4.5	-12.7 ± 3.5	-5.8 ± 1.4	0.26
	**120 min**	9.2 ± 2.2	2.4 ± 1.6	0.5 ± 2.5*	0.03
	**240 min**	3.7 ± 1.8	1.5 ± 1.4	-0.5 ± 2.3	0.31
**SER**	**Fasting**	0.04 ± 0.04	0.03 ± 0.04	0.06 ± 0.02	0.15
	**120 min**	0.07 ± 0.02	0.04 ± 0.05	0.08 ± 0.02	0.88
	**240 min**	0.08 ± 0.02	0.02 ± 0.04	0.08 ± 0.02	0.57
**SGU (µmol/kg/min)**	**Fasting**	2.9 ± 5.5	3.7 ± 5.5	4.6 ± 2.6	0.97
	**120 min**	12.9 ± 4.8	6.2 ± 9.0	8.0 ± 2.8	0.70
	**240 min**	13.5 ± 4.4	6.5 ± 8.4	7.7 ± 2.4	0.61
**SGP (µmol/kg/min)**	**Fasting**	-16.0 ± 3.7	-16.4 ± 7.9	-10.4 ± 3.1	0.64
	**120 min**	-3.8 ± 3.2	-3.8 ± 9.3	-7.5 ± 4.7	0.87
	**240 min**	-9.8 ± 4.9	-5.1 ± 8.4	-8.2 ± 2.8	0.84

Supplementary Table 1: Net Splanchnic Glucose Balance (NSGB), Splanchnic Extraction Ratio (SER), Splanchnic Glucose Uptake (SGU) and Splanchnic Glucose Production during fasting and the two stages of the clamp. Data represent Mean ± SEM. *P*-values represent results of a one-way analysis of variance (ANOVA) test. **Post-hoc* Tukey's test suggests a significant difference for the Obese groups vs. the Lean group.

Leucine influx (L1) = C_ALeu x F
Leucine efflux (L2) = C_VLeu x F
Leucine uptake = L1 – L2

KIC Influx (K1) = C_AKIC x F
KIC Efflux (K2) = C_VKIC x F
KIC release = K2-K1

Leucine N flux = L1 x ($E_A D_{Leu}/E_V D_{Leu}$-1)
Leucine C flux = L1 x (E_ALeu/E_VLeu-1)
Reamination (T2) = (N_{flux}-C_{flux})/(1-(E_VLeu-E_VKIC)/E_VLeu)
Breakdown (B) = N flux – T2

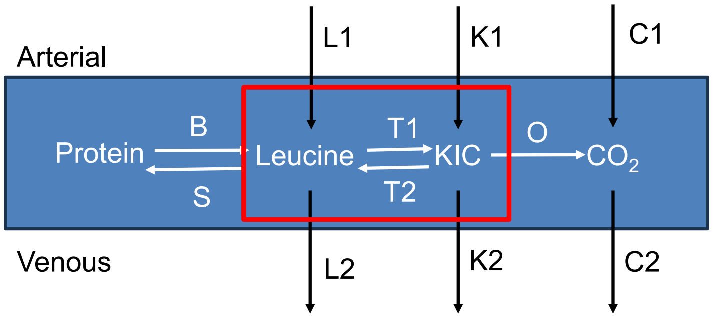

Supplementary Figure 4: Equation(s) used to calculate Leucine metabolism across the splanchnic tissues using the model described by Cheng, K. N., et al. (1985). Direct determination of leucine metabolism and protein breakdown in humans using L-[1-^{13}C, ^{15}N]-leucine and the forearm model. *Eur J Clin Invest* **15**(6): 349-354. Leucine uptake is shown in Figure 3, Panel A. α-ketoisocaproic acid (KIC) release is shown in Figure 3, Panel B. Reamination is shown in Figure 3, Panel C and leucine breakdown (B) is shown in Figure 3, Panel D. C_A and C_V = arterial and venous concentrations respectively. E_A and E_V = arterial and venous enrichment respectively. F = splanchnic plasma flow.

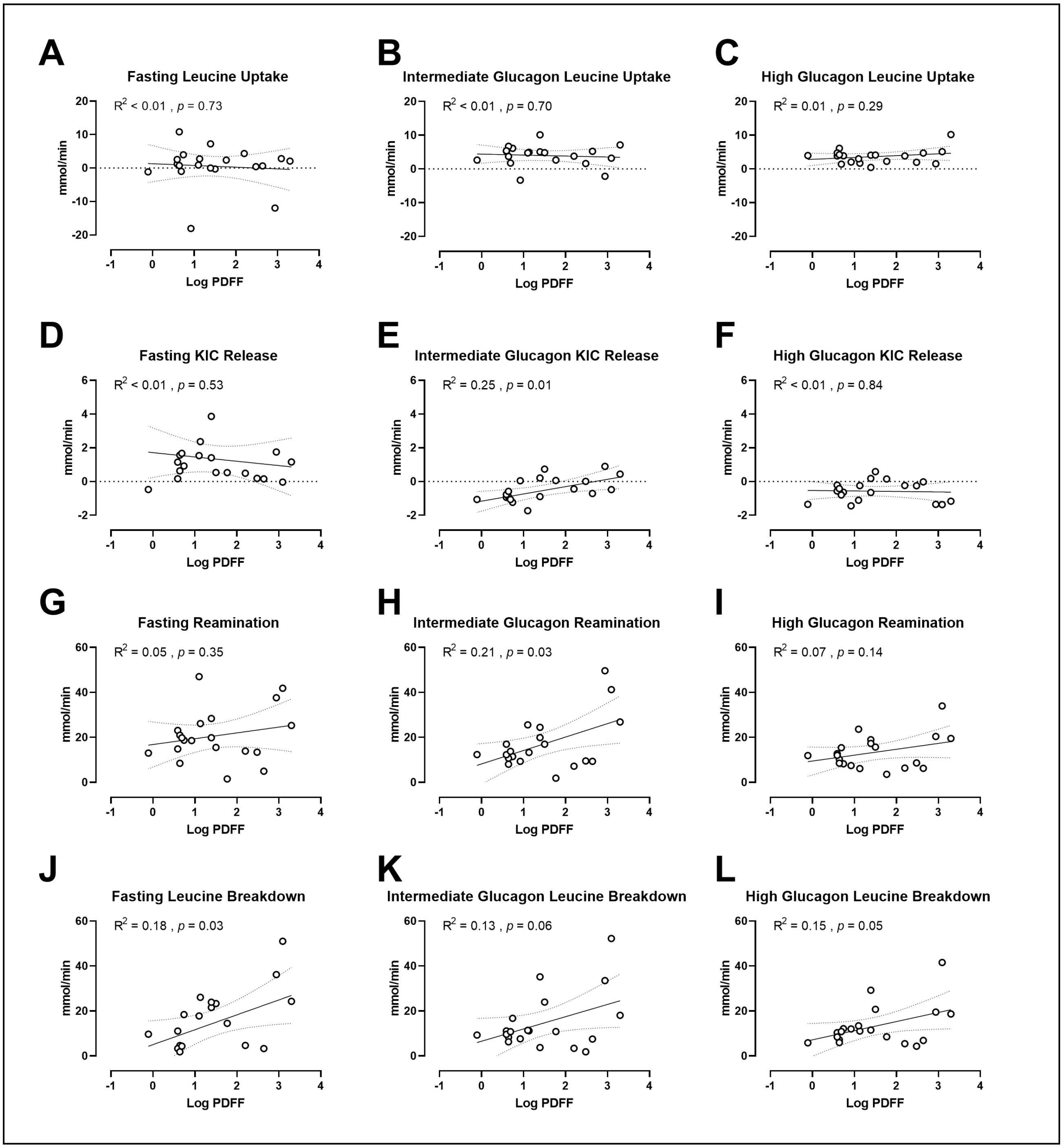

Supplementary Figure 5: Simple linear regression for the relationship of leucine metabolism with (log-transformed) hepatic Proton Density Fat Fraction (PDFF) during fasting (0 min), intermediate glucagon infusion (120 min) and during high glucagon infusion (240 min). KIC = α-ketoisocaproic acid.

$$Splanchnic\ Balance = (AA_{FA} - AA_{HV}) \times SPF$$

$$Splanchnic\ Balance = (Metabolite_{FA} - Metabolite_{HV}) \times SPF$$

Supplementary Figure 6: Equation(s) used to calculate Splanchnic Balance of a given Amino Acid or Metabolite. FA = Femoral Artery; HV = Hepatic Vein; SPF = Splanchnic Plasma Flow.

Splanchnic Balance of	Time (min)	PDFF (%)		Weight (Kg)	
		R^{2*}	*P*-value	R^{2*}	*P*-value
**Alanine**	0	< 0.01	0.56	0.01	0.09
	120	< 0.01	0.44	0.08	0.12
	240	< 0.01	0.91	0.02	0.24
**Arginine**	0	0.05	0.18	< 0.01	0.71
	120	**0.24 (-0.49)**	**0.02**	< 0.01	0.92
	240	**0.18 (-0.42)**	**0.04**	< 0.01	0.59
**Cysteine**	0	< 0.01	0.47	0.07	0.14
	120	< 0.01	0.34	0.06	0.16
	240	< 0.01	0.49	0.06	0.16
**Glutamate**	0	< 0.01	0.98	**0.22 (-0.47)**	**0.02**
	120	< 0.01	0.67	0.09	0.11
	240	< 0.01	0.81	0.11	0.08
**Glutamine**	0	< 0.01	0.37	< 0.01	0.67
	120	< 0.01	0.89	**0.19 (0.44)**	**0.03**
	240	< 0.01	0.86	< 0.01	0.43
**Glycine**	0	< 0.01	0.75	0.06	0.16
	120	0.06	0.16	**0.35 (0.59)**	**0.01**
	240	0.08	0.12	**0.52 (0.72)**	**0.01**
**Methionine**	0	0.04	0.20	0.01	0.29
	120	< 0.01	0.32	0.06	0.16
	240	< 0.01	0.67	0.06	0.16
**Serine**	0	0.04	0.22	< 0.01	0.56
	120	< 0.01	0.32	< 0.01	0.42
	240	< 0.01	0.36	< 0.01	0.34
**Threonine**	0	0.02	0.24	< 0.01	0.53
	120	< 0.01	0.31	0.02	0.25
	240	< 0.01	0.71	< 0.01	0.40
**Tyrosine**	0	< 0.01	0.40	0.02	0.24
	120	< 0.01	0.50	0.07	0.14
	240	< 0.01	0.75	0.02	0.25

Supplementary Table 2: Relationship of splanchnic balance for specific amino acids and metabolites with hepatic fat as quantified by the Proton Density Fat Fraction (PDFF), and with body weight. Significant correlations are in **bold** type. *Where significant, R-values are provided in parentheses to show the direction of the correlation.

Splanchnic Balance of	Time (min)	PDFF (%)		Weight (Kg)	
		R^{2*}	*P*-value	R^{2*}	*P*-value
**allo-isoleucine**	0	< 0.01	0.56	< 0.01	0.85
	120	< 0.01	0.44	0.03	0.22
	240	**0.24 (0.49)**	**0.02**	< 0.01	0.50
**α-aminoadipic acid**	0	< 0.01	0.30	0.12	0.07
	120	< 0.01	0.52	0.08	0.12
	240	< 0.01	0.40	**0.27 (-0.52)**	**0.01**
**α-amino-N-butyric acid**	0	< 0.01	0.82	< 0.01	0.49
	120	< 0.01	0.89	< 0.01	0.31
	240	0.02	0.25	0.04	0.19
**β-alanine**	0	< 0.01	0.72	**0.19 (0.44)**	**0.03**
	120	< 0.01	0.87	**0.34 (0.58)**	**< 0.01**
	240	< 0.01	0.99	**0.18 (0.42)**	**0.04**
**β-amino-isobutyric acid**	0	**0.38 (0.62)**	**< 0.01**	< 0.01	0.58
	120	**0.34 (0.58)**	**< 0.01**	< 0.01	0.93
	240	**0.31 (0.56)**	**< 0.01**	< 0.01	0.84
**Citrulline**	0	0.05	0.18	< 0.01	0.40
	120	**0.20 (0.45)**	**0.03**	< 0.01	0.86
	240	**0.19 (0.44)**	**0.03**	< 0.01	0.47
**Ethanolamine**	0	0.10	0.10	< 0.01	0.70
	120	**0.28 (-0.53)**	**< 0.01**	< 0.01	0.79
	240	0.14	0.06	< 0.01	0.85
**γ-amino-N-butyric acid**	0	**0.18 (-0.42)**	**0.04**	< 0.01	0.37
	120	**0.15 (-0.39)**	**0.05**	< 0.01	0.68
	240	**0.25 (-0.50)**	**0.01**	0.02	0.27
**Hydoxylysine**	0	< 0.01	0.74	< 0.01	0.53
	120	**0.24 (-0.49)**	**0.02**	< 0.01	0.25
	240	< 0.01	0.89	**0.22 (-0.47)**	**0.02**

Supplementary Table 3: Relationship of splanchnic balance for specific amino acid metabolites and related metabolites with hepatic fat as quantified by the Proton Density Fat Fraction (PDFF), and with body weight. Significant correlations are in **bold** type. *Where significant, R-values are provided in parentheses to show the direction of the correlation.

$$\text{Extraction of an Amino Acid} = \frac{[Amino\ Acid]_{FA} - [Amino\ Acid]_{HV}}{[Amino\ Acid]_{FA}}$$

$$\text{Extraction of a Metabolite} = \frac{[Metabolite]_{FA} - [Metabolite]_{HV}}{[Metabolite]_{FA}}$$

Supplementary Figure 7: Equation(s) used to calculate Splanchnic Extraction Ratio of a given Amino Acid or Metabolite. FA = Femoral Artery; HV = Hepatic Vein.

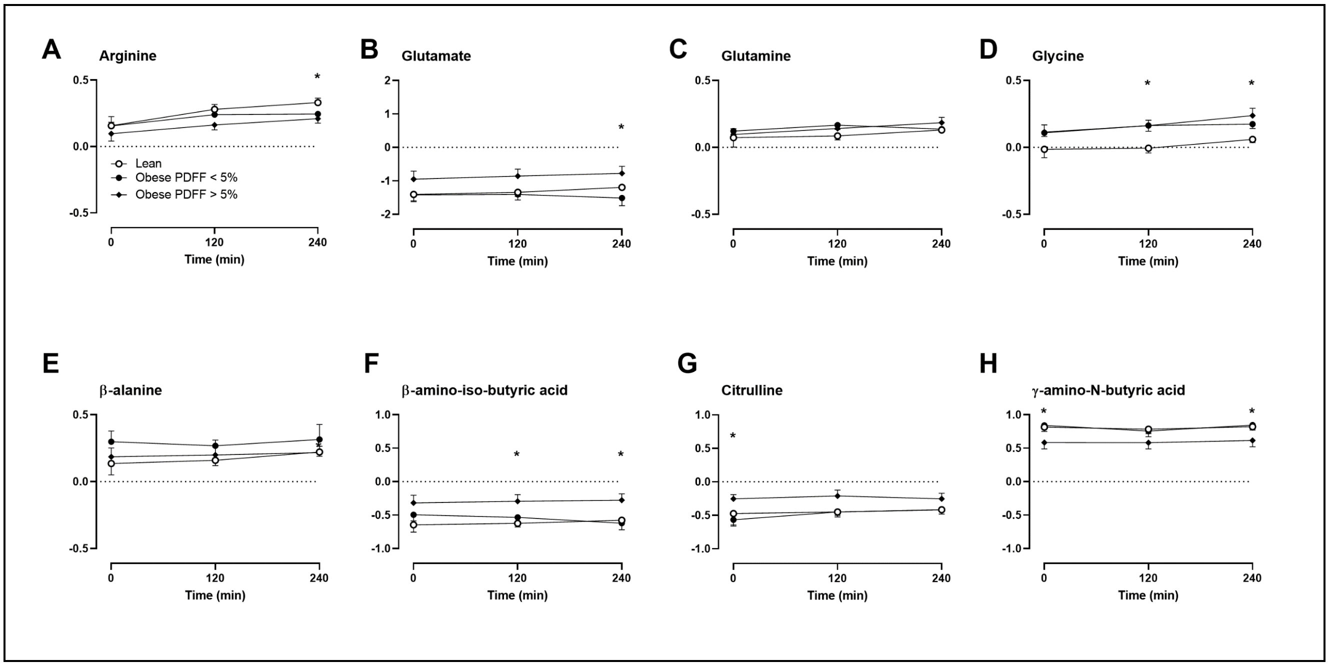

Supplementary Figure 8: Splanchnic Extraction Ratio for selected Amino Acids (Panels A to D) and metabolites (Panels E-H) at fasting (0 min) and the intermediate and high glucagon infusion rates (120 min and 240 min, respectively) of the clamp in lean subjects (○), obese subjects with a PDFF < 5% (●) and obese subjects with a PDFF > 5% (♦). * * *p*-value < 0.05 for a one-way analysis of variance (ANOVA) test; PDFF = Proton Density Fat Fraction. n = 7 in the Lean group, n = 6 in the obese group with PDFF < 5% and n = 7 in the obese group with PDFF > 5%.
